# Perivascular epithelioid cell tumor of the lung: A case report and literature review

**DOI:** 10.1111/1759-7714.14583

**Published:** 2022-07-24

**Authors:** Shaofu Yu, Shasha Zhai, Qian Gong, Xiaoping Hu, Wenjuan Yang, Liyu Liu, Yi Kong, Lin Wu, Xingxiang Pu

**Affiliations:** ^1^ Department of Clinical Pharmacy the Second People's Hospital of Huaihua Huaihua Hunan China; ^2^ The Second Department of Thoracic Medical Oncology Hunan Cancer Hospital Changsha Hunan China; ^3^ Department of Trauma Surgery The First Affiliated Hospital of Hunan University of Medicine Huaihua Hunan China; ^4^ Department of Clinical Pharmacy Hunan Cancer Hospital Changsha Hunan China; ^5^ Department of Pathology Hunan Cancer Hospital Changsha Hunan China; ^6^ Department of Radiotherapy Hunan Cancer Hospital Changsha Hunan China

**Keywords:** CCST, clear cell sugar tumor, PEComa, perivascular epithelioid cell tumor, lung

## Abstract

The perivascular epithelioid cell tumor (PEComa) is a rare tumor of interstitial origin characterized by several immunological and muscle cell markers. The clear cell sugar tumor (CCST) of the lung is a type of PEComa defined by thin cell walls and high levels of glycogen in the cytoplasm. We herein reported the case of a 48‐year‐old male with a recurrence of lung CCST. The preoperative diagnosis of the lung mass was performed by percutaneous needle biopsy. During the thoracoscopic resection, multiple adhesions in the thoracic cavity were described. The tumor invaded the chest wall, and the boundaries between the tumor and surrounding normal tissues were unclear. The mediastinal lymph nodes were significantly enlarged. No relevant gene mutations were detected. After concomitant chemoradiotherapy, the patient's condition was stable. We also conducted a literature review and discussed the overall findings.

## Graphical abstract

In this work, we reported a rare case of perivascularepithelioid cell tumor (PEComa) of the lung, and also conducted a systematicliterature review and discussion. The overall efficacy of the patient wasevaluated as stable disease (SD) after systemic therapy, and the patient'scondition was stable during continuous outpatient follow‐up.

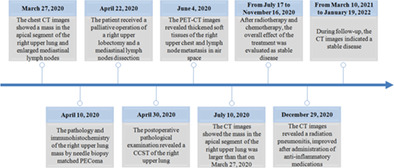

## INTRODUCTION

The perivascular epithelioid cell tumor (PEComa) is a very rare mesenchymal neoplasm characterized by specific histological and immunohistochemical features.^1^ A primary PEComa commonly occurs in the uterus, kidney and liver, but not in the lung. The clear cell sugar tumor (CCST) is a subtype of PEComa^2^ that was first described in the lung by Liebow and Castleman in 1963.^3^ This study reports a single case of lung CCST observed at the Second Department of Thoracic Oncology of Hunan Cancer Hospital (China). We also conducted a literature review and discussed the overall findings.

## CASE PRESENTATION

**FIGURE 1:**
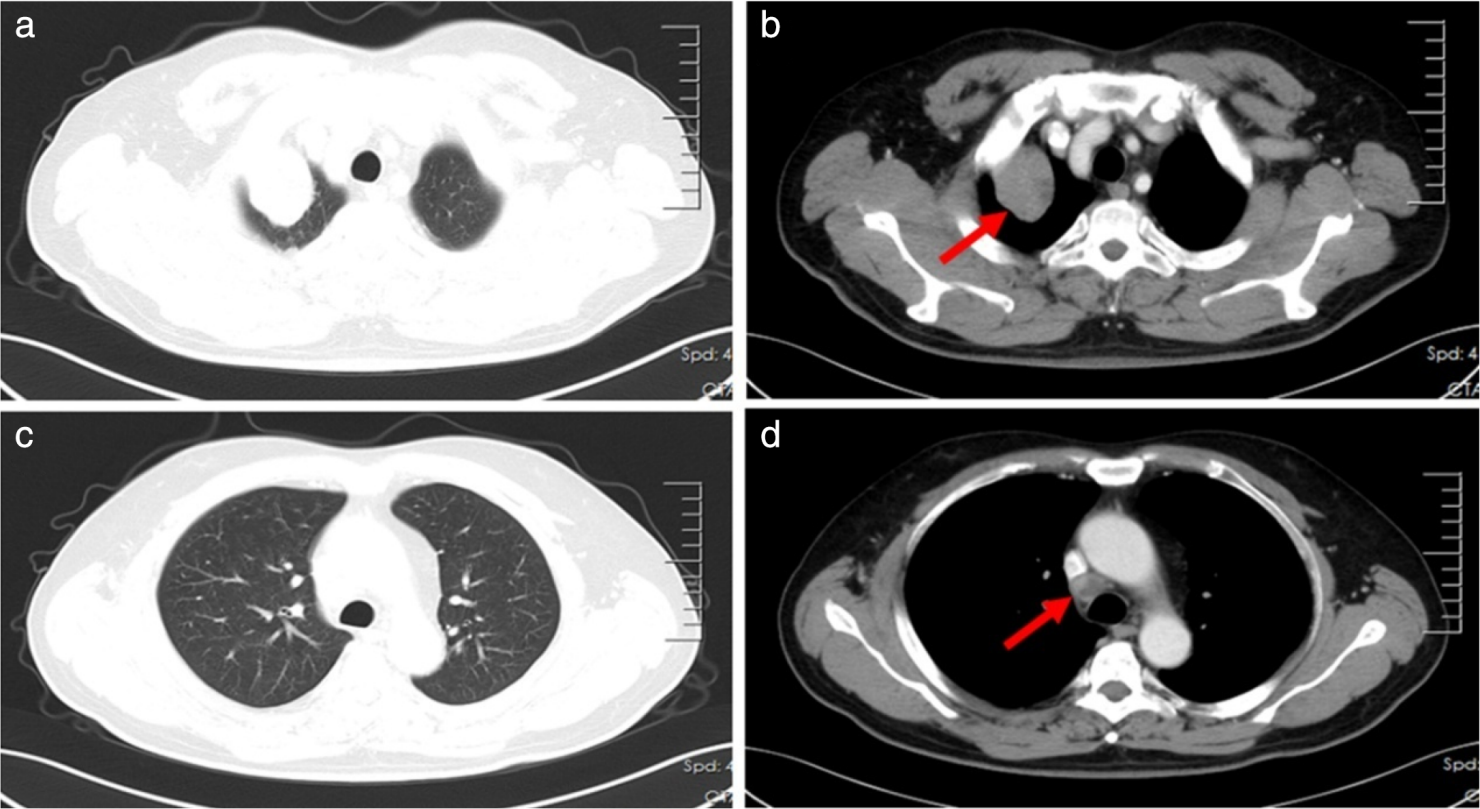
Chest CT images showed a mass in the apical segment of the right upper lung and enlarged mediastinal lymph nodes

**FIGURE 2:**
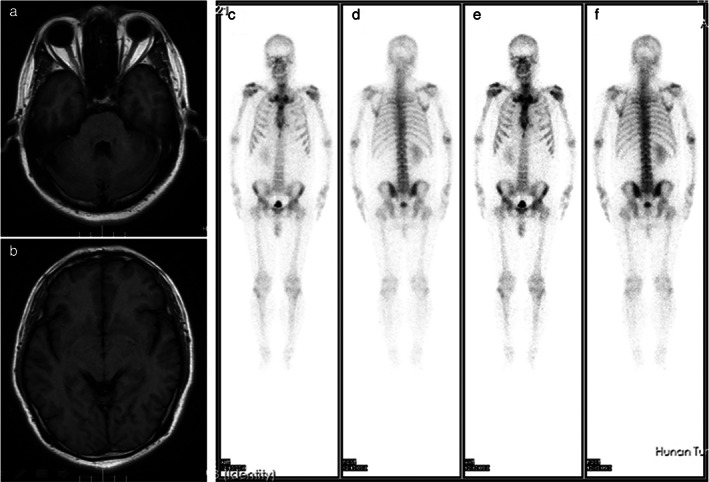
No abnormalities were reported with bone single‐photon emission computed tomography or brain magnetic resonance imaging

**FIGURE 3:**
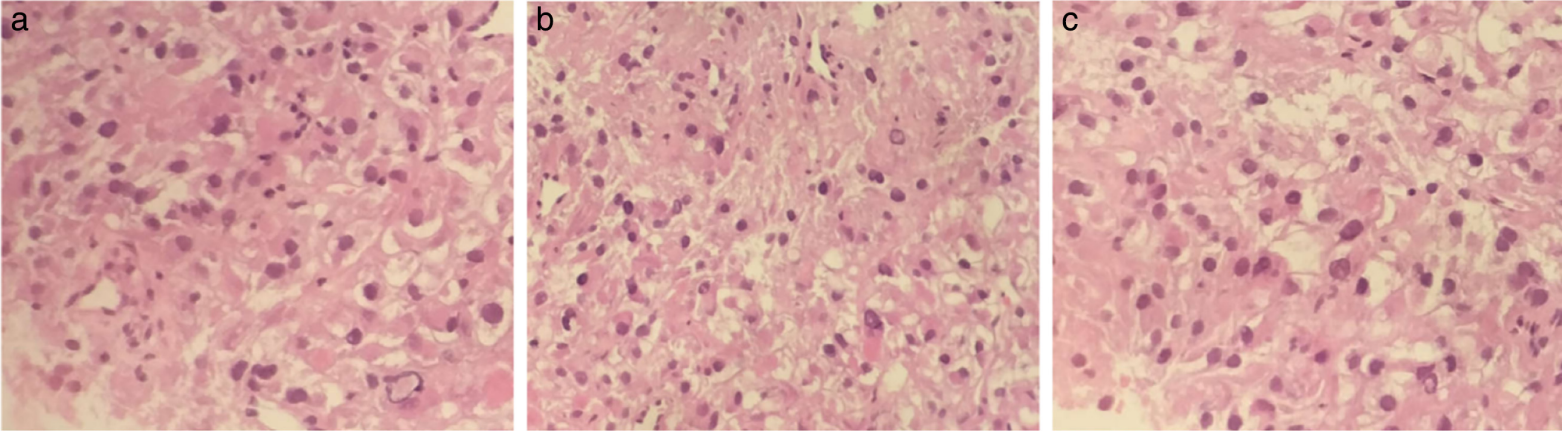
The needle biopsy of the lung mass showed an epithelioid tumor

A 48‐year‐old man was admitted at the Second Department of Thoracic Oncology of Hunan Cancer Hospital on March 25, 2020 due to a lung mass. The lung mass was diagnosed 1 month before. The chest computerized tomography (CT) performed on March 27, 2020 showed enlarged mediastinal lymph nodes and a mass in the apical segment of the right upper lobe (Figure 1). No abnormalities were found using bone single‐photon emission computed tomography (ECT), brain magnetic resonance imaging (MRI) (Figure 2), and tumor markers. The needle biopsy pathology of the lung mass performed on April 10, 2020 revealed an epithelioid tumor (Figure 3). The immunohistochemical evaluation showed a positive immunoreactivity for PNL‐2, MelanA, SMA, HMB45, and VIM, but not for SOX‐10, S‐100, CK, LCK, CK7, CK5/6, P40, TTF‐1, Napsin A, Syn or CgA, which matched a PEComa.

On May 10, 2007, the patient underwent a resection of a left renal angiomyolipoma (AML). He reported a history of hypertension for 4 years, regularly treated with nifedipine (20 mg oral tablets, once per day). He reported no history of smoking, alcohol consumption or tumor‐related family history.

There were no contraindications to surgery at the preoperative examinations. The patient received surgical treatment on April 22, 2020. The mass (size 5.0 × 4.0 × 4.0 cm) in the right upper lung invaded the chest wall with no clear boundaries to the vessels. The mediastinal lymph nodes were enlarged. There was no pleural effusion. No other nodules were observed in the remaining lung parenchyma. During the surgical operation, the gross specimens were removed and the adhesion between the lung mass and the chest wall was relieved. The lymph nodes in groups 3, 7, 8, 10 and 11 were cleaned. The lymph nodes in groups 2 and 4 were enlarged and invaded the superior vena cava, thus part of them were removed. The residual tumor tissues were cauterized and R2 resection was performed. After resection, the PEComa was diagnosed as stage IIIB (pT3N2M0).

**FIGURE 4:**
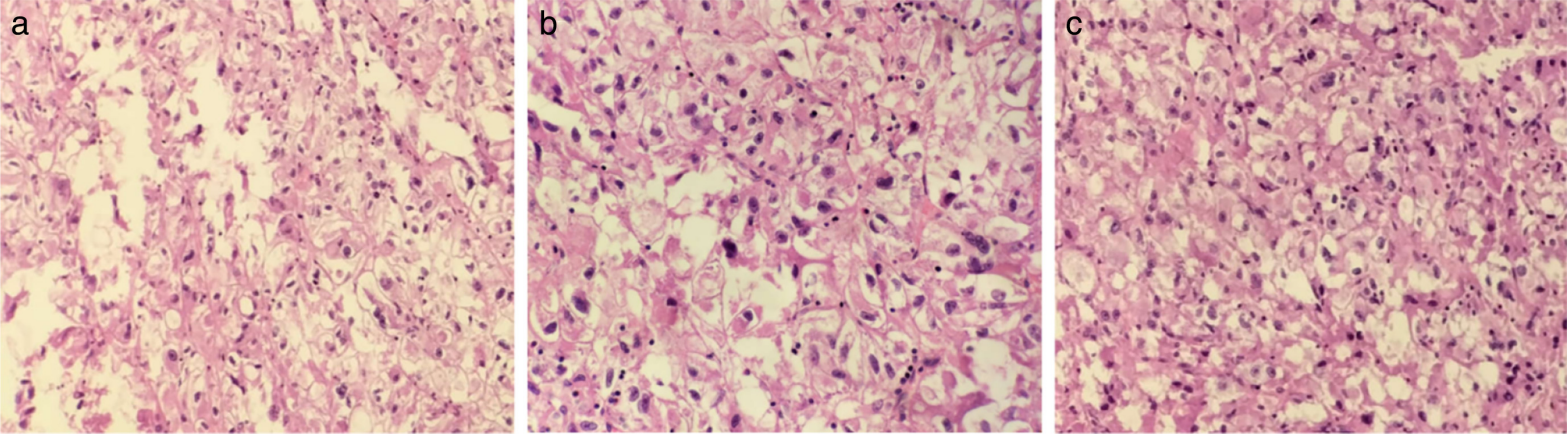
Postoperative examination revealed a CCST of the right upper lung

The postoperative pathological examination performed on April 30, 2020 (Figure 4) revealed a CCST of the right upper lung. The tumor was a solitary nodule without an envelope, located at the periphery of the right upper lung. It was composed of large cells with clear cytoplasm, with eosinophilic granules containing glycogen. The nuclei were round or oval, centered and hyperchromatic, without mitotic figures. Most of the tumor cells were distributed in sheets around thin‐walled vessels. The perivascular interstitium was characterized by hyaline degeneration or calcification. The expression of programmed cell death ligand 1 (PD‐L1) was negative.

On June 4, 2020, the positron emission tomography‐computed tomography (PET‐CT) revealed thickened soft tissues of the right upper chest. The positron emission tomography (PET) found clumps of abnormal radioactive concentration in the corresponding area, suggesting the presence of a residual tumor. A nodular soft tissue density shadow was observed in the air space. An abnormal radioactive concentration shadow was observed in the corresponding area, suggesting the presence of lymph node metastases. There were no other abnormalities in the chest.

**FIGURE 5:**
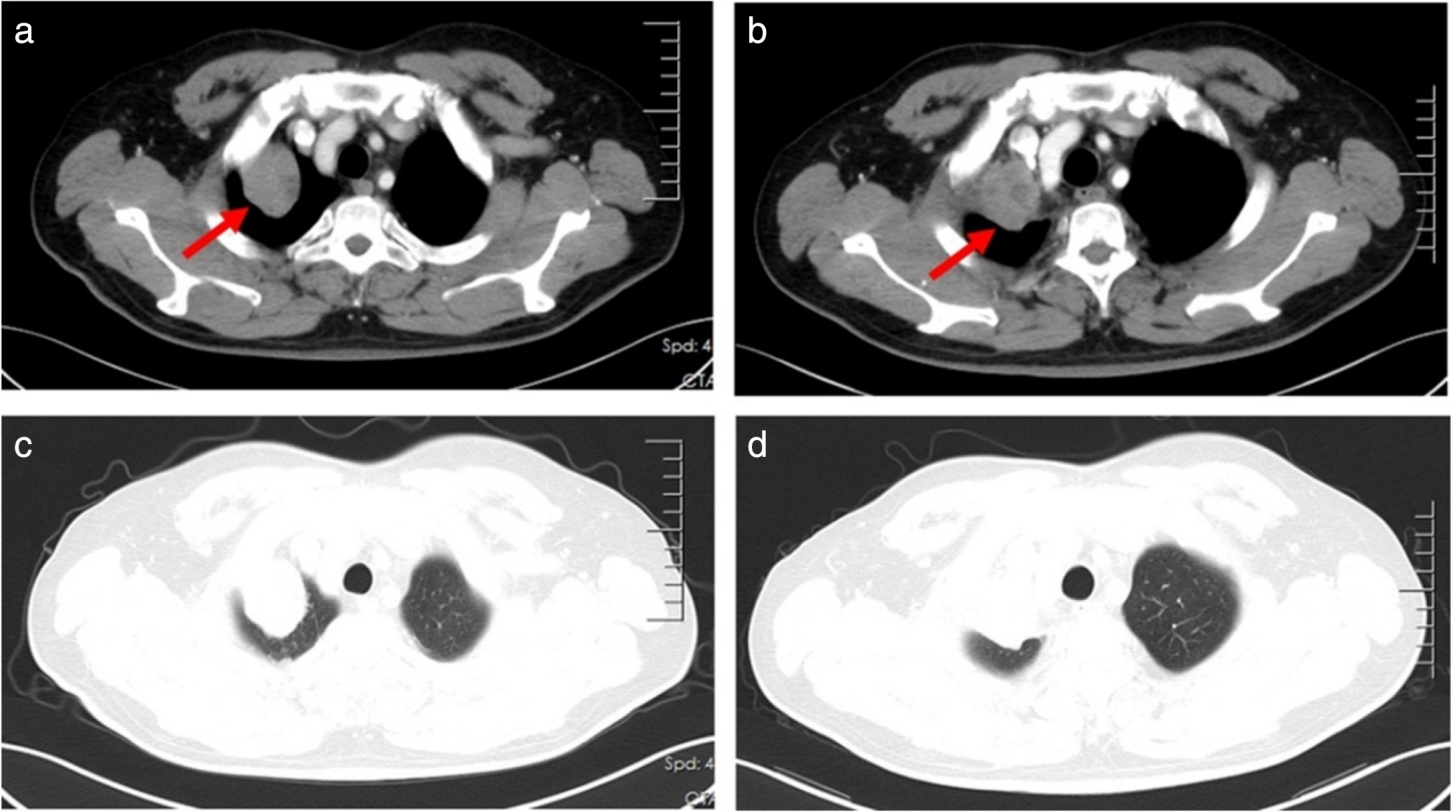
The comparison of CT images obtained on July 10, 2020 (b and d) and March 27, 2020 (a and c) showed a larger mass in the apical segment of the right upper lung. The mediastinal lymph nodes in group 4R were enlarged as before. Small nodules appeared in both lower lungs

The comparison of CT images between July 10, 2020 and March 27, 2020 showed a larger mass in the apical segment of the right upper lung. The mediastinal lymph nodes in group 4R were enlarged as before. Small nodules appeared in both lower lungs, which were considered tumor recurrence (Figure 5).

**FIGURE 6:**
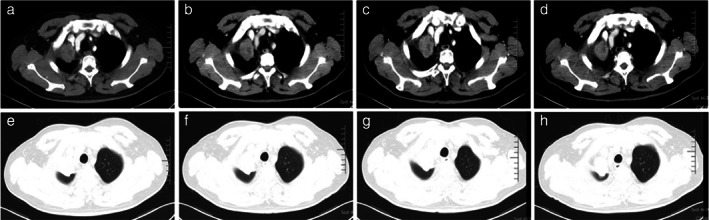
After radiotherapy and chemotherapy, the overall effect of the treatment was evaluated as stable disease by comparing CT images acquired on July 10 (a and e), August 7 (b and f), September 29 (c and g), and November 16, 2020 (d and h)

The patient received three cycles of chemotherapy, paclitaxel (albumin bound) 400 mg combined with cisplatin 130 mg on July 17, August 8, and August 30, 2020. Due to a gastrointestinal reaction, the patient was subsequently treated with paclitaxel (albumin bound) 400 mg combined with carboplatin 450 mg for three more cycles on September 30, October 26, and November 16, 2020. The patient received three‐dimensional conformal intensity modulated radiotherapy from July 21 to August 28, 2020. The overall effect was evaluated as stable disease (Figure 6).

**FIGURE 7:**
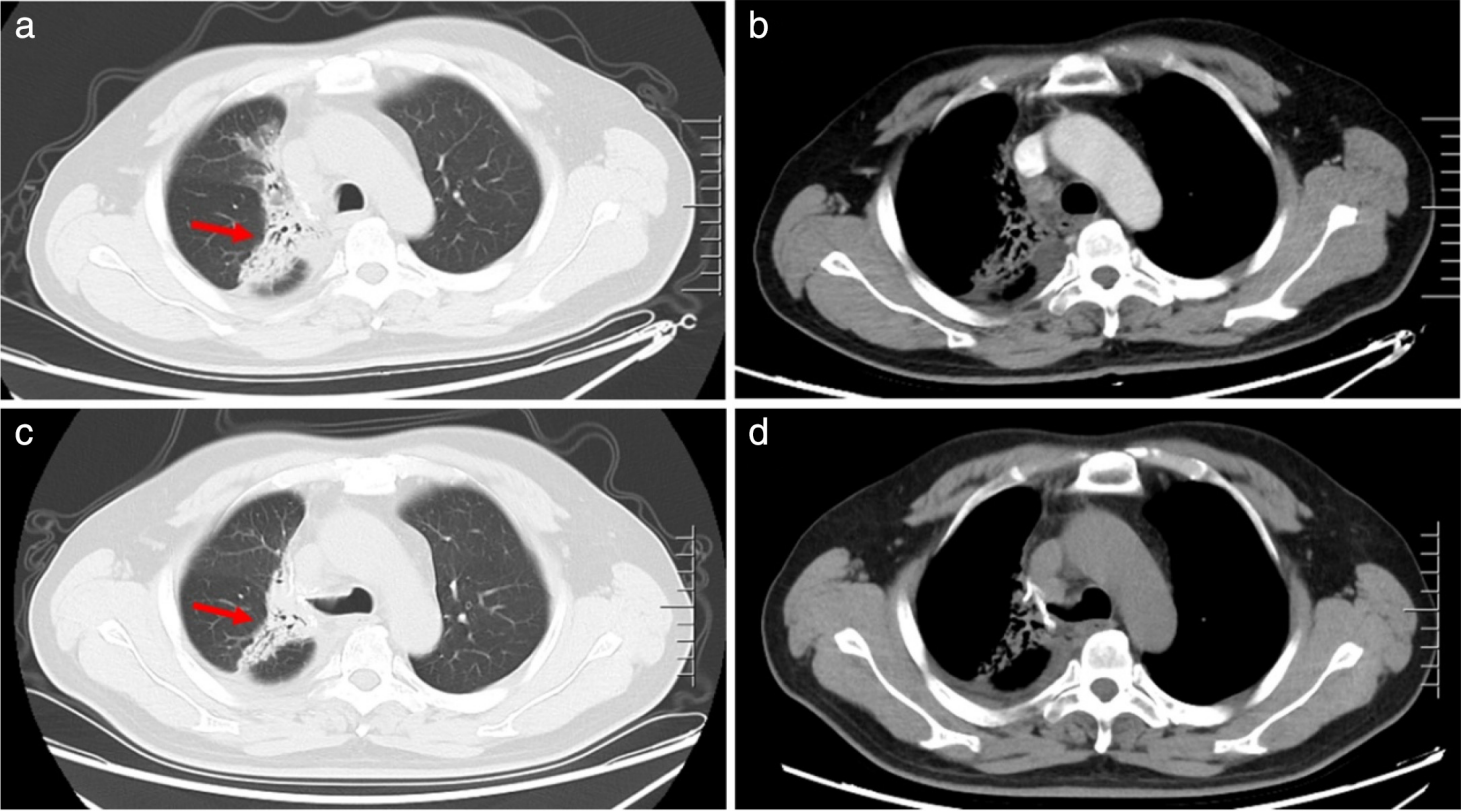
The patient presented with cough and chest pain on December 29, 2020. The CT images (a and b) revealed a radiation pneumonitis, improved after administration of anti‐inflammatory medications (c and d)

**FIGURE 8:**
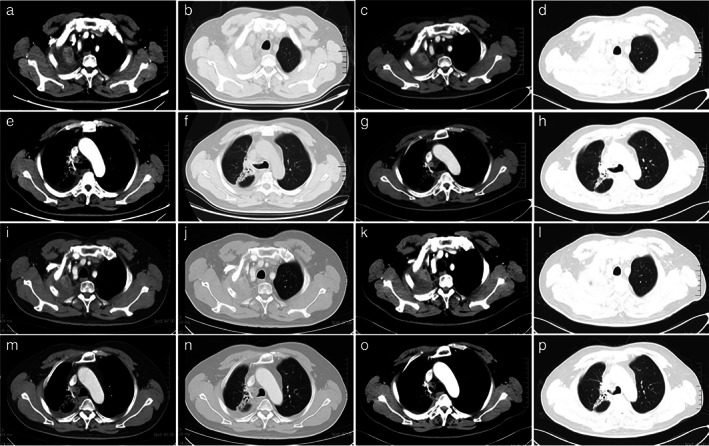
The CT images indicated a stable disease among March 10 (a, b, e, and f), June 10 (c, d, g, and h), September 16, 2021 (i, j, m, and n), and January 19, 2022 (k, l, o, and p)

**FIGURE 9:**
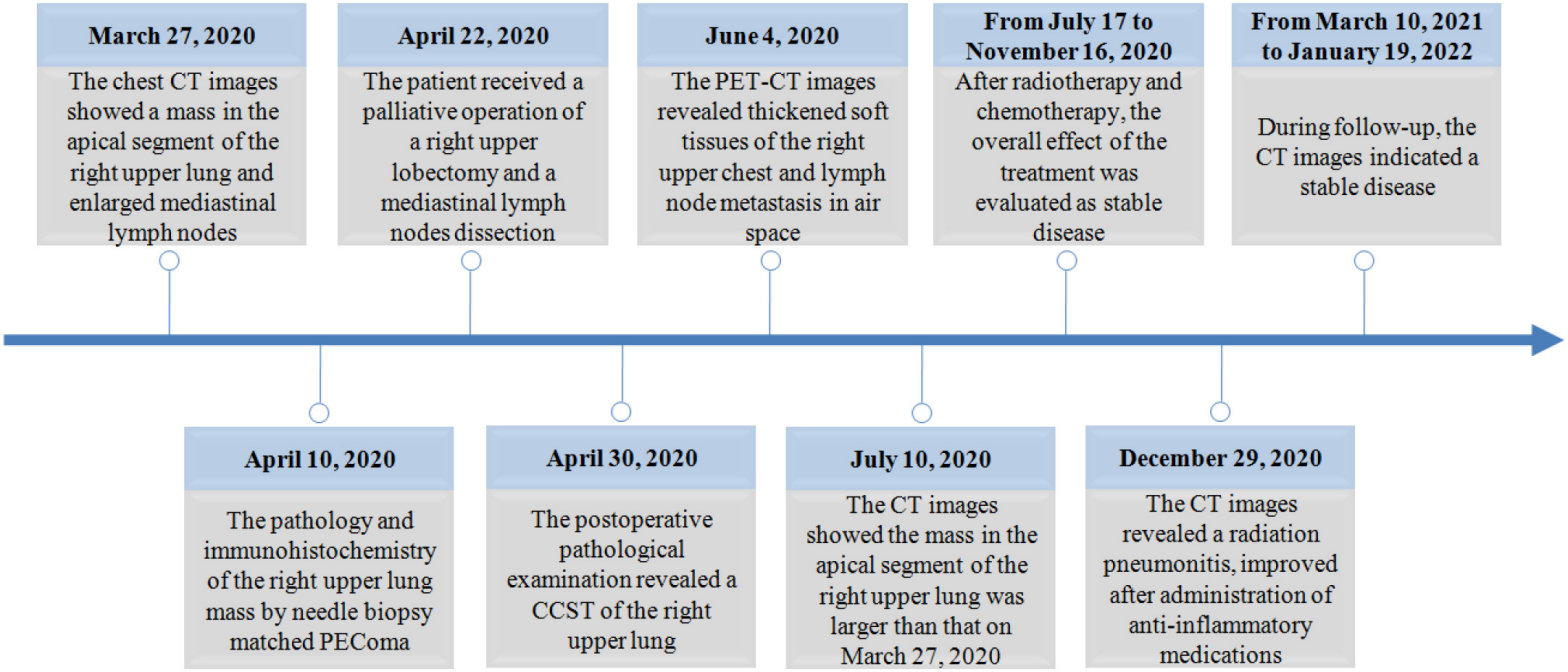
The diagnostic and treatment history of the patient

On December 29, 2020, the patient presented with cough and chest pain. The CT images revealed a radiation pneumonitis, improved after administration of anti‐inflammatory medications (Figure 7). The CT images indicated a stable disease between March 10, 2021 and January 19, 2022 (Figure 8). The diagnostic and treatment history of the patient is described in Figure 9.

## DISCUSSION

PEComas are mesenchymal tumors radially arranged around thick‐walled vessels. They are characterized by cells with clear to weakly eosinophilic, granular cytoplasm, mainly expressing melanin markers, such as HMB45, and myogenic markers, such as SMA. PEComas include a series of tumors, such as angiomyolipoma, lymphangioleiomyomatosis, clear cell sugar tumor, and malignant PEComas.^2^ The types and features of PEComas are described in Table 1.

**TABLE 1:** Types and features of PEComas

PEComas	Location
Angiomyolipoma Common subtypes: classic type, epithelioid type, lipomatoid type, myxomatous type Rare subtypes: epithelial cyst type, eosinophilic tumor type, sclerosis type	Usually occurring in kidneys and other organs
Lymphangioleiomyoma Lymphangioleiomyomatosis	Usually occurring in lungs, followed by other organs
Clear cell sugar tumor	Usually occurring in lungs
Malignant PEComa	Rare

We searched PubMed, Embase, the Cochrane Library, the China National Knowledge Infrastructure, the Wanfang Data, and the China Science and Technology Journal database with the search terms “perivascular epithelioid cell tumor”, “PEComa”, “clear cell sugar tumor” and “CCST” from inception to January 22, 2022. We retrieved 29 case reports^4^, ^5^, ^6^, ^7^, ^8^, ^9^, ^10^, ^11^, ^12^, ^13^, ^14^, ^15^, ^16^, ^17^, ^18^, ^19^, ^20^, ^21^, ^22^, ^23^, ^24^, ^25^, ^26^, ^27^, ^28^, ^29^, ^30^, ^31^, ^32^ of primary lung PEComa, which are summarized in Table 2. They included 12 female patients and 17 male patients, ranging from 28 to 79 years old (mean age 54 years). Tumors ranged from 0.7 to 18.0 cm in diameter, with an average of 4.3 cm. Fourteen patients reported symptoms, including cough, chest tightness, chest pain, and hemoptysis. The other patients were asymptomatic. Eight tumors were malignant and the remaining 21 cases were benign. All patients underwent surgical resection except one patient who refused treatment. Four patients reported coexisting tumors: a tumor with 15% adenocarcinoma features, a single adenocarcinoma in a different lobe of the same lung, a single mediastinal PEComa, and a single metachronous hepatic angiomyolipoma. Six patients did not report the follow‐up situation. In the other cases, 19 patients reported no recurrence, three patients had recurrence or metastases, and two patients died before the corresponding studies were published. One patient died of respiratory failure and the other one died of cardiopulmonary failure.

**TABLE 2:** Summary of previous reports regarding primary lung PEComa

First author (publication year)	Gender	Age	Tumor location	Tumor size (cm)	Symptoms	Pathology	Immunohistochemistry	Coexisting tumors	Resectability	Treatment	Prognosis
ZH Wang (2021)^4^	F	56	The lower lobe of the left lung	6.2 × 4.5 cm	NM	CCST	Positive for HMB45, MelanA, CD34, and CD10; negative for PCK, EMA, CK8/18, SMA, DES, Caldesmon, S‐100, SOX10, and PAX8; the Ki‐67 score was about 2%	No	Yes	Left lower lung mass resection with lymph node dissection	No recurrence or metastasis in the 6 months after surgery
HJ Huang (2021)^5^	M	46	The lingual segment of the left upper lobe, partly invading the basal segment of the lower lung, adjacent to the pleura, the anterior and posterior thoracic wall and the diaphragm	Lung mass with a size of 17.0 × 14.0 × 6.0 cm; the other mass with a size of 11.0 × 7.0 × 6.0 cm at 2.0 cm from the incision margin of the lung bronchus and immediately adjacent to the visceral pleura	Cough and chest pain for more than 10 days	Malignant PEComa (85%) and adenocarcinoma, acinar subtype (15%)	Positive for VIM, HMB45, and TFE3; negative for CK, CD34, S‐100, SMA, and desmin; the p53 gene mutations were about 90%; the Ki‐67 score was about 90%; a small number of tumor cells forming a cribriform or glandular tubular shape were described with moderate atypia, positive for CK7 and TTF‐1; negative for CK5/6 and P40; the Ki‐67 score was about 15%	Adenocarcinoma, acinar subtype (15%)	Yes	Left upper lung mass resection with lymph node dissection; six cycles of chemotherapy (epirubicin combined with ifosfamide) and afatinib targeted therapy for EGFR sensitive mutation	Stable condition at 6 months
LL Shen (2020)^6^	F	28	The lower lobe of the left lung	About 49 nodules, the largest was around 2.8 cm in diameter	Left chest paroxysmal pricking	CCTL	Positive for HMB45, CD34, and VIM; negative for cytokeratin, SMA, S‐100, CD10, PAX‐8, desmin, and Myo‐D1	No	Yes	Left lower lobectomy and mediastinal lymph node dissection	No metastasis or recurrence after 6 months
M Wang (2019)^7^	M	61	The lower lobe of the left lung	0.7 × 0.7 cm	The left lower lung nodules were associated with no symptoms	PEComa	Positive for CD34, HMB45, MelanA, S‐100, and SMA; negative for AE1/3; the Ki‐67 score was about 2%	No	Yes	Left lower lobe lobectomy and lymph node dissection	The patient is still followed up
JK Zhao (2019)^8^	M	54	The middle lobe of the right lung	About 4.0 cm in diameter	NM	Malignant PEComa	Positive for VIM, MelanA, and TFE3; negative for HMB45; the Ki‐67 score was about 10%	Primary adenocarcinoma of the lower lobe of the left lung	Yes	Tumor dissection of the middle lobe of the right lung, wedge‐resection of the lower lobe of the left lung and lymph node dissection; three cycles of chemotherapy (paclitaxel combined with carboplatin)	Stable conditions
M Sjniari (2019)^9^	M	74	The apical portion of the right lung	About 2.8 cm in diameter	NM	CCST	Positive for CD10, pan‐CK, MNF116, and CK7; negative for TTF‐1	No	Yes	Right lobectomy and mediastinal lymphadenectomy	No recurrence or metastasis after 4 years
EK Yeon (2018)^10^	M	58	The lower lobe of the right lung	About 2.7 cm in diameter	NM	CCST	Positive for HMB‐45, VIM, and CD34; negative for AE1/3 and EMA	No	Yes	Wedge resection of the lower lobe of the right lung	No recurrence or metastasis after surgery
DI Tsilimigras (2018)^11^	M	46	The upper and middle lobe of the right lung	About 5.5 cm in diameter	Without symptoms of cough, hemoptysis, shortness of breath or voice hoarseness	CCST	Positive for HMB45, MART‐1, SMA, and desmin; negative for AE1/3, CK‐7, CK20, and EMA	No	Yes	Right middle lobectomy and anterior upper segmentectomy	NM
M Chang (2018)^12^	F	61	The upper lobe of the left lung	3.0 × 2.5 × 2.5 cm	Without symptoms of cough, hemoptysis or shortness of breath	CCST	Positive for HMB‐45 and CD34; negative for S‐100, AE1/3, SMA, calponin, GFAP, desmin, TTF‐1, P40, and PAX‐8	No	Yes	Thoracoscopic surgery wedge resection of the tumor	Stable conditions
YH Song (2017)^13^	F	49	The lower lobe of the right lung	4.0 × 3.0 × 2.0 cm	Cough and chest pain	CCST	Positive for HMB‐45, MelanA, CD34, CD1a, and SMA; negative for CK, Syn, chromogranin, S‐100, TTF‐1, SP‐A, CD31, desmin, mucin, CK7, and CD117; the Ki‐67 score was about 3–5%	No	Yes	Surgical thoracoscopic right lower lobectomy	No metastasis or recurrence after 6 months
A Chakrabarti (2017)^14^	M	36	The upper and middle lobe of the right lung	18.0 × 13.0 cm	Right‐sided chest pain for 2 months and a history of hemoptysis	Malignant PEComa	Positive for TFE‐3, desmin, and SMA; negative for CK, EMA, CD68, HMB‐45, MelanA, S‐100, myogenin, and MiTF	No	Yes	Right upper and middle lobectomy	A sensation of heaviness in the right thorax for 6 months after surgery; the CT images of the thorax showed a lung mass extending into the lower part of the neck up to the posterior paravertebral soft tissues, with erosion of the upper ribs and a metastatic lesion in the right head of the humerus
XY Shi (2016)^15^	F	50	The lower lobe of the left lung	Nodules of different sizes diffusely distributed in both lungs, with evident exudative shadows	Cough and dyspnea for 60 days, hemoptysis for 40 days and fever for 7 days	Malignant PEComa	Positive for HMB45, VIM, and SMA; negative for MelanA, CDX‐2, CD56, Syn, CgA, CK7, Napsin A, TTF‐1, EMA, and CD10; the Ki‐67 score was about 25%	No	NA	Declined further specific therapy	Rapid progressive respiratory failure, the patient died 2 weeks after the diagnosis
HY Kim (2016)^16^	M	51	The upper lobe of the right lung	About 1.0 cm in diameter	Without symptoms	PEComa	Positive for HMB‐45 and MelanA; negative for S‐100, CD56, Syn, CgA, TTF‐1, surfactant, Napsin A, and cytokeratins; the Ki‐67 score was below 2%	No	Yes	Wedge resection of the upper lobe of the right lung	NM
HB Sun (2015)^17^	F	78	The lower lobe of the right lung	3.0 × 2.5 × 2.5 cm	Without symptoms of cough, hemoptysis, chest pain, chest tightness or fever	CCST	Positive for VIM, Bcl‐2, CD34, and MelanA; negative for CK, HMB‐45, SMA, and S‐100; the Ki‐67 score was about 1%	No	Yes	Thoracoscopic mass resection	No metastasis or recurrence after 6 months
WJ Liang (2015)^18^	M	63	The upper lobe of the left lung and the anterior mediastinum	Left upper lobe mass with a size of 4.2 × 4.7 cm; mediastinal mass with a size of 6.7 × 9.8 cm	Chest pain for more than 2 months	Malignant PEComa	Surgical specimens from the left lung masses: positive for VIM, HMB45, and MelanA; negative for PAN‐CK, EMA, and S‐100 Surgical specimens from the mediastinum masses: positive for TFE3, VIM, MelanA, and HMB45; negative for P63, SMA, CK7, CD10, PAX8, PAN‐CK, Napsin A, and TTF‐1	Mediastinal PEComa	Yes	Resection of the tumor in the left upper lung and mediastinum	The anterior mediastinal mass recurred, the metastatic tumor in the left rib enlarged in 3 months after surgery; the patient died of cardiopulmonary failure approximately 7 months after surgery
AH Olivencia‐Yurvati (2015)^19^	F	39	The upper lobe of the left lung	1.1 × 1.0 cm	NM	CCST	NM	No	Yes	Wedge resection of the upper lobe of the left lung	NM
S Neri (2014)^20^	M	38	The middle lobe of the right lung	1.8 × 1.5 × 1.3 cm	NM	CCST	Positive for HMB‐45, VIM, and SMA; negative for S100, desmin, AE1/3, EMA, and CD117	AML of the liver in 2005	Yes	Wedge resection of the middle lobe of the right lung	No metastasis or recurrence after 13 months
L Deng (2013)^21^	F	54	The lower lobe of the right lung	5.0 × 4.0 × 4.0 cm	Cough, hemoptysis, and chest tightness for more than 2 months	Malignant PEComa	Positive for HMB45, PNL2, and A013; negative for AEl/3, CAM5.2, and VIM	No	Yes	Resection of right lower lobe mass and mediastinal lymphadenectomy	The patient is still followed up, without metastasis
GX Wang (2013)^22^	M	38	The lower lobe of the left lung	About 3.4 cm in diameter	Recurrent cough, blood‐streaked sputum for 2 months, and left chest pain for 10 days	CCST	Positive for HMB45, VIM, CD34, and S‐100; negative for CK, desmin, CD68, EMA, RCC, and TTF‐1	No	Yes	Wedge resection of the lower lobe of the left lung	No metastasis or recurrence after 12 months
B Yan (2011)^23^	F	75	The lower lobe of the left lung	2.8 × 2.2 × 2.0 cm	Fever of unknown origin for 3 months	CCST	Positive for S‐100, HHF35, HMB45, and VIM; negative for AE1/3, EMA, SMA, desmin, CD34, NSE, CgA, and Syn	No	Yes	Resection of left lower lobe mass	No metastasis or recurrence after 10 years
ZY Wang (2010)^24^	M	79	The lower lobe of the left lung	5.0 × 3.0 × 3.0 cm	Cough and sputum for 1 week	Malignant PEComa	Positive for HMB45 and VIM; negative for LCA, CD138, S‐100, CD34, EMA, CK, TTF‐1, Syn, and NSE; the Ki‐67 score was about 50%	No	Yes	Resection of left lower lobe and mediastinal lymphadenectomy; one cycle of chemotherapy (gemcitabine) after surgery, not continued because of poor tolerance	Extensive metastasis of both lungs, left pleura and lymph nodes at 3 months after surgery; the patient refused further treatment. The patients is alive with no clear symptoms at 5 months after surgery
T Ye (2010)^25^	F	50	The lower lobe of the right lung	About 4.0 cm in diameter	A sensation of chest tightness for almost 2 months	Malignant CCST	Positive for HMB45, PNL2, and A013; negative for VIM, AE1/3, and CAM5.2	No	Yes	Resection of right lower lobe and mediastinal lymphadenectomy	NM
S Sen (2009)^26^	F	44	The upper lobe of the right lung	4.0 × 3.0 cm	Headache and weakness	CCST	Positive for S‐100 and HMB45; negative for CK and CD68	No	Yes	Resection of the tumor at the right upper lung	No complication or recurrence occurred in the postoperative period
HF Gu (2008)^27^	F	54	The lower lobe of the right lung	About 3.5 cm in diameter	Without symptoms	PEComa	Positive for HMB45, CD34, S‐100, and Actin; negative for CK and EMA	No	Yes	Resection of right lower lung	No recurrence
WJ Kim (2008)^28^	M	64	The upper lobe of the left lung	1.2 × 1.0 cm	NM	CCST	Positive for HMB45 and S‐100; negative for CK	No	Yes	Wedge resection of the tumor at the left upper lung	No metastasis or recurrence after 2 months
ML Policarpio‐Nicolas (2008)^29^	M	64	The lateral basilar segment of the right lobe	2.2 × 2.0 × 1.9 cm	Shortness of breath on exertion	CCST	Positive for HMB45 and MelanA; negative for EMA, AE1/3, RCC, and S‐100	No	Yes	Wedge resection of the tumor at the right lobe	NM
B Papla (2003)^30^	M	68	The superior segment in the lower lobe of the right lung	About 1.2 cm in diameter	Without symptoms	CCST	Positive for HMB45, NSE, S‐100, and ACT; negative for TTF‐1, CgA, and CD117	No	Yes	Wedge resection of a fragment of the right lower lobe	The postoperative course is without complications
ZH Ding (1996)^31^	M	34	The posterior segment in the upper lobe of left lung	3.0 × 3.0 × 3.6 cm	Chest tightness, chest pain, and cough for 3 months	CCST	NM	No	Yes	Resection of left upper lobe mass	NM
WP Harbin (1978)^32^	M	65	The lower lobe of the right lung	About 2.0 cm in diameter	Denied hemoptysis, sputum production, fever or weight loss	CCST	NM	No	Yes	Resection of left lower lobe	No metastasis or recurrence after 18 months

Abbreviations: AML, angiomyolipoma; CCST, clear cell sugar tumor; F, female; M, male; NA, not available; NM, not mentioned; PEComa, perivascular epithelioid cell tumor.

CCST is a rare tumor characterized by transparent cells rich in cytoplasm and glycogen.^33^ CCST usually occurs in middle‐aged or elderly people, with no significant difference in incidence between men and women. It usually occurs in lungs, followed by other organs. CCST patients are usually asymptomatic, and only a few manifest chest pain and cough. CCST is usually found by accident in the lungs, like a “coin”, usually isolated and clearly defined without impairing the pleura.^28^, ^29^, ^34^

According to the pathology, CCST generally distributes like nests or flakes, with clear boundaries between the tumor and the surrounding areas. Fibrous intervals are described between nests, with tumor cells arranged radially around blood vessels. In general, immunohistochemical markers of CCST are positive for HMB45, MelanA, and SMA and negative for CK and CD10.^35^

The main treatment of CCST is surgical resection, with postoperative follow‐up.^36^ Some tumors that cannot be surgically removed underwent postoperative adjuvant radiotherapy and chemotherapy. Some CCST patients are complicated with tuberous sclerosis complex (TSC), TSC1/2 gene mutation, and activation of the mTOR signal transduction pathway, therefore mTOR inhibitors such as everolimus or sirolimus might be used for treatment.^37^, ^38^, ^39^

## CONCLUSION

CCST of the lung belongs to a rare subtype of PEComa, generally isolated and clearly defined, which usually occurs in middle‐aged or elderly people. Neoplastic cells are transparent and characterized by cytoplasm rich in glycogen. CCST patients usually report no symptoms, with only a few complaining of chest pain and cough. The main treatment is a complete surgical resection, with postoperative follow‐up. Adjuvant radiotherapy and chemotherapy can be used when necessary. Patients with TSC1/2 gene mutation are sensitive to mTOR inhibitors, including everolimus and sirolimus.

In our case report, the patient performed an R2 resection. The surface of the residual tumor was cauterized. Two months after surgery, the tumor reappeared in the right upper apical segment of the lung. It was slightly larger than before. After adjuvant radiotherapy and chemotherapy, the overall efficacy was evaluated as stable disease. There were no abnormalities regarding tumor markers. The radiotherapy caused a radiation pneumonia, which improved after treatment with anti‐inflammatory medications. During follow‐up, the CT images indicated a stable disease.

## FUNDING INFORMATION

This work was funded by the Beijing CSCO Clinical Oncology Research Foundation (No: Y‐XD202001‐0215) and the Hunan Provincial Natural Science Foundation of China (No: 2019jj80018).

## CONFLICT OF INTEREST

The authors report no conflicts of interest related to this study.

## CONSENT FOR PUBLICATION

A written informed consent was obtained from the patient for publication of his medical history and relative records. No information that would enable his identification has been provided.

## AUTHORS' CONTRIBUTIONS

S.Y., S.Z., Q.G., L.L., and Y.K. contributed to the design of the study, and the acquisition and analysis of data. S.Y. drafted and wrote the manuscript. X.H., W.Y., L.W., and X.P. critically reviewed the manuscript. All authors read and approved the final version of the manuscript.

## ETHICAL APPROVAL

The study protocol was approved by the Ethics Committee of Hunan Cancer Hospital (Appendix S1).

## Supporting information


**Appendix S1** Supporting Information.
