# Supplementary figures and images for: Perivascular epithelioid cell tumor of the lung: A case report and literature review
Source: Thorac Cancer. 2022 Jul 24;13(17):2542–53. doi: 10.1111/1759-7714.14583 (PMC9436685; doi:10.1111/1759-7714.14583)

**Ethical approval information**


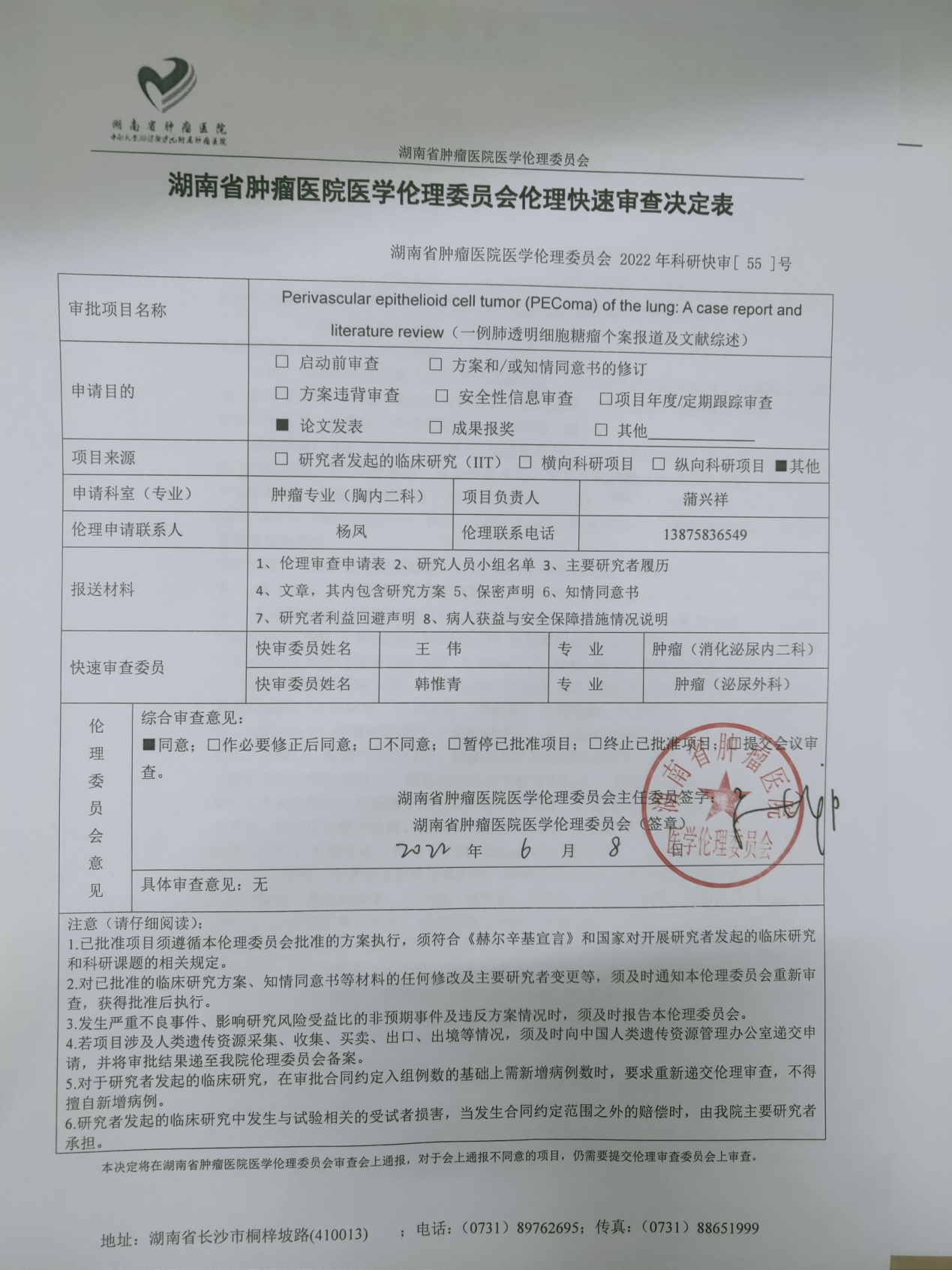

Supplement: Supplementary file 1 — Appendix S1 Supporting Information. [file TCA-13-2542-s001.docx]
